# Do’s and Don’ts of Taking Care of Deaf Patients

**DOI:** 10.21980/J8336T

**Published:** 2025-01-31

**Authors:** Luke Johnson, Sarah Smetana, Wyatte Hall, Aaron D Weaver, Jason Rotoli

**Affiliations:** * University of Rochester School of Medicine and Dentistry, Strong Memorial Hospital, Department of Emergency Medicine, Rochester, NY; ^ University of Rochester Medical Center, Strong Memorial Hospital, Department of Public Sciences, Rochester, NY

## Abstract

**Audience:**

Emergency medicine residents, fellows, and attending physicians, any practicing provider in a medical setting that may serve Deaf patients.

**Introduction:**

Emergency medicine providers often interact with Deaf and Hard of Hearing (DHH, or just HOH, for only hard of hearing) patients. Various limitations, however, affect their ability to effectively engage with DHH patients such as acuity, lack of time, and/or readily available communication tools (eg. virtual or in-person interpreters), among other challenges. These barriers contribute to numerous DHH healthcare disparities. Estimating the number of DHH people and ASL users in the US is challenging because the US Census Bureau inquires about hearing loss as it (1) pertains to interactions between a person speaking and the person (who may be experiencing hearing loss or deafness) being spoken to and (2) does not inquire if ASL is used in the home as a primary language.^1^,^2^ In reviewing data from the 2002 Survey of Income and Program Participation (SIPP), there were approximately 11 million people (4.1%) in the US with hearing loss and 1 million (0.38%) who are functionally deaf (unable to hear “normal” conversation at all).^2^ Best estimates of the number of *total people* using sign language in the US come from survey data from the National Census of the Deaf Population in 1974.^3^ In this survey, it was noted that approximately 410,522 people have been signing in homes irrespective of hearing status (i.e. may include signing to hearing household members of DHH family). In considering prevocational deaf individuals (i.e. born deaf or lost the ability to hear before 19 years old), there are approximately 277,000 deaf people who are considered “good signers.”^4^ Understanding that the DHH community makes up an important portion of our patient population, we sought to design an educational intervention and infographic to demonstrate common pitfalls while caring for this marginalized group in the Emergency Department (ED). Not only does this community face difficulties navigating the health care system due to communication barriers and poor health literacy, but DHH and American Sign Language (ASL) users also appear to have higher rates of ED utilization than the general population of non-DHH individuals.^5^,^6^ Despite increased ED utilization, disparities persist such as extended door-to-disposition time, limited diagnostic studies, lack of IV placement, and lower likelihood of hospital admission.^7^,^8^ Our project sought to help mitigate these disparities by engaging a group of highly dedicated individuals seeking to improve the quality of care for DHH patients in our community. Collectively, we developed an instructional video and quick reference infographic to help educate providers in preferred communication strategies and in pitfalls to avoid while communicating with DHH patients.

**Educational Objectives:**

By the end of this didactic, the learner will demonstrate increased comfort with communication with DHH patients via improved awareness of communication pitfalls and through approaches to communicating with DHH patients in a limited capacity, such as without timely access to interpreters or in an environment where staff are unfamiliar with DHH patients. An in-depth assessment of cultural awareness and description of proper communication techniques, necessary equipment, or interpreter working relationships is beyond the scope of this project.

**Educational Methods:**

A video entitled, “Do’s and Don’ts of Taking Care of Deaf Patients,” including still shots from the video and a Word document containing an infographic with QR code to the educational video. Instead of a static PowerPoint presentation, we simulated and recorded a low-fidelity clinical scenario in order to better mirror a real-life scenario. Additionally, the use of multimedia in education (and motivating instructional features such as graphics/scenarios/videos) has been shown to increase satisfaction and generative processing in comparison to reading alone.^9^,^10^

**Research Methods:**

This project was undertaken as a quality improvement (QI) initiative, and as per the University of Rochester’s Guideline for Determining Human Subject Research, it did not meet the definition of research according to 45CFR46 and was exempt from IRB approval. This QI project employed the Plan-Do-Study-Act (PDSA) strategy in which we used a pre- and post-survey to assess the impact of our intervention on participant knowledge of Deaf culture and self-reported comfort in caring for DHH individuals.^11^ In order to engage key stake holders and best design our project, we collaborated with a group of highly dedicated individuals seeking to improve the quality of care for DHH patients. These individuals included ASL interpreters, hospital staff, DHH medical students, DHH community members, and emergency medicine faculty and residents. Collectively, and through an iterative process, we were able to capitalize on the QI team and key stakeholders’ expertise to design the initial and follow up surveys. The QI team refined the surveys and subsequently pre-tested them for errors and comprehension. We did not formally assess construct validity, but the survey was reviewed by content experts and key stakeholders for face validity. We surveyed EM attending physicians, residents, fellows, and advanced practice providers (APP) within our department. A pre-intervention email with a Redcap survey link was sent to the EM resident, fellow, faculty, and APP listservs with a subsequent in-person reminder within our didactic conference and two weeks later to maximize participation.^12^ After pre-intervention responses were gathered and analyzed, a link to a simulated patient care educational video was distributed through the same listservs with a post-intervention survey. Two weeks later, after post-intervention data analysis and part of the second PDSA cycle, an educational quick reference infographic was created and distributed to help further educate providers in communicating effectively with DHH patients. Here we present the educational content created as a result of this collaboration. For this project, we used means and frequencies as descriptive statistics to characterize the sample. Due to the small sample size and nature of the QI project, statistical significance was not assessed.

**Results:**

The pre-intervention survey included responses from 50 individuals including 25 residents (50%), 5 medical students (10%), and 20 faculty (40%). The post-intervention survey included 26 responses including 13 residents (50%), 2 medical students (10%), and 11 faculty (60%). Though statistical significance was not assessed due to sample size, in general, there was a trend toward improved comfort with communication in all areas of interest. Questions were answered either by a Likert scale, true/false, or multiple choice. There was an increase in the percentage of providers being “mostly” or “completely comfortable” in obtaining a history without an interpreter (18% vs 29%), obtaining a history with an interpreter (80% vs 96%), performing a procedure (76% vs 92%), and delivering a diagnosis or patient counseling (76% vs 97%). The average pre-intervention knowledge score was 75% (standard deviation of 25) and the post-intervention knowledge score was 85 % (standard deviation of 16).

**Discussion:**

We demonstrated self-reported improvements in comfort for nearly every aspect of patient care after an educational intervention, which is consistent with previous Deaf cultural awareness training results.^13^,^14^ It will be important to assess in the future if improved provider comfort in working with DHH patients translates to an improvement in the DHH patient experience. Additionally, DHH cultural knowledge increased during our QI project potentially allowing for increased cultural awareness. Collectively, increased comfort and cultural awareness allows providers to deliver more equitable care to a marginalized group. Important lessons learned during the creation of this video and subsequent infographic are (1) that it is feasible (and necessary) to produce educational materials with the input of members of the Deaf culture and (2) that there is a need for enhanced provider understanding of Deaf culture to take care of Deaf patients. In our PDSA survey, we measured an improvement in self-reported comfort and understanding of Deaf culture with our intervention. There were limitations of our QI project. We did have participant attrition often seen in cross-sectional survey-based data collection. Also, results should be interpreted with caution since statistical significance was not calculated due to the nature of the QI project and to the small number of participants. It is important to note that our participants had a high level of baseline comfort with obtaining a history with an interpreter (compared to obtaining it without) due to the increased availability of interpreter resources in our institution. While we are fortunate to have this resource, this may not be a common finding and thus limit generalizability to other institutions with less interpreter service availability.

**Conclusion:**

Through watching an educational video of a clinical scenario and reviewing this infographic, providers may have improved awareness of communication pitfalls to avoid and some strategies to use while caring for DHH patients in the ED.

**Topics:**

Health inequities, health disparities, disability, education, advocacy.

## USER GUIDE

**Table:** 

List of Resources:	
Abstract	1
User Guide	4
Do’s and Don’ts of Taking Care of Deaf Patients Lecture and Handout	6

**Learner Audience:**

Medical Students, Interns, Junior Residents, Senior Residents, All Providers, but we believe learners should be prioritized first to engage in early habits.

**Time Required for Implementation:** 30 minutes

**Topics:**

Health inequities, health disparities, disability, education, advocacy.

**Objectives:**

By the end of this lecture, the learner will be able to:

1. Demonstrate basic approaches to communicating with Deaf and hard-of-hearing patients in a limited capacity, such as without timely access to interpreters or in an environment with staff unfamiliar with DHH patients.
2. Describe common pitfalls to avoid when caring for DHH patients

### Linked objectives and methods

This handout is derived from a rigorous quality improvement project within a three-year emergency medicine residency program that serves a large DHH and ASL-using population. Baseline understanding of resident comfort, confidence, and awareness of Deaf culture was quantified with a preeducational intervention survey, followed by an educational video, and subsequent post-survey analysis of knowledge changes. Our intervention survey is based on a prior study by Hoang^13^ where the competence of Deaf culture was assessed at an institution with Deaf culture education. With permission from the authors, we edited this study with further feedback from Deaf academic faculty, medical professionals, and students.

During our Plan-Do-Study-Act (PDSA) cycle,^15^ this educational intervention showed some improvement. We subsequently designed an infographic highlighting pitfalls and solutions to further improve understanding of DHH culture and communication strategies. Our overall design plan then was to create a project using two forms of easily accessible and understandable media for multiple reasons, including 1) delivery of concise and engaging information to busy individuals, 2) free access to information, 3) spaced repetition to improve memory with an easily referenceable infographic for repeated exposure, 4) ability to disseminate widely across departments or even hospitals nationwide, and 5) clear demonstration of teaching points to improve in clarity while also showing real-life consequences (eg, poor patient outcome in our video). Designing this project as easily consumable material highlights its ability to be disseminated and repeatedly referenced as a powerful agent to promote retention for eventual use in clinical practice. Our format has been designed to achieve clarity, accessibility, and a thought-provoking reminder of how poor outcomes can be perpetuated if providers do not have some cultural awareness of the patient population for whom they provide care.

### Link to Lecture

- https://youtu.be/qaRPuvOx_KI

### Results and tips for successful implementation

Our total number of survey responses (n) started at 50 respondents and decreased to 26 respondents. Reasons for attrition may include confusion regarding completion of the online survey or survey fatigue.^16^,^17^ While we did not assess for statistical significance due to small sample size and the nature of the QI project, we saw general trends in improvement of comfort and objective DHH culture knowledge (75% average correct pre-intervention vs 85% average correct post-intervention) for caring for DHH patients based on our pre-video, post-video, and pre-infographic surveys. These were the main outcomes of the project and demonstrates the success of this QI initiative. In designing our infographic, we included highlights from the educational video, engaging photos, and QR code to serve as a quick reference guide for clinicians.

In the future, ways this project could improve is to focus on survey delivery in a more structured setting such as in a classroom over a few hours with other lectures in between rather than over email and multiple days. Another way we hope to improve this overall project and benefit to the Deaf and HOH community would be to assess the comfort of Deaf and HOH patients with these providers after exposure to this new tool. When using this tool either with residents or as required reading during a faculty meeting, one could assess the patient’s general comfort level or feeling of being understood after a visit to the emergency room. Or, on a more systems level, one could assess the number of complaints from Deaf and HOH patients who comment on not having access to interpreters during their visit.

### Technology necessary

Camera/phone, computer to run YouTube, or at least a video with high definition.

## Supplementary Information









## Figures and Tables

**Figure:**
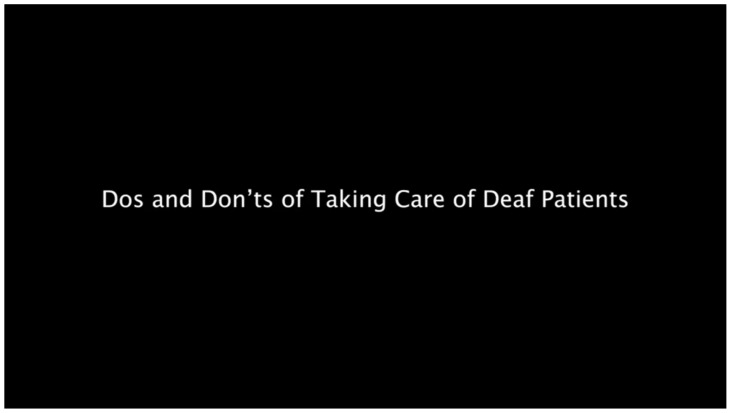
Please see associated video file Video Link: https://youtu.be/qaRPuvOx_KI

**Figure:**
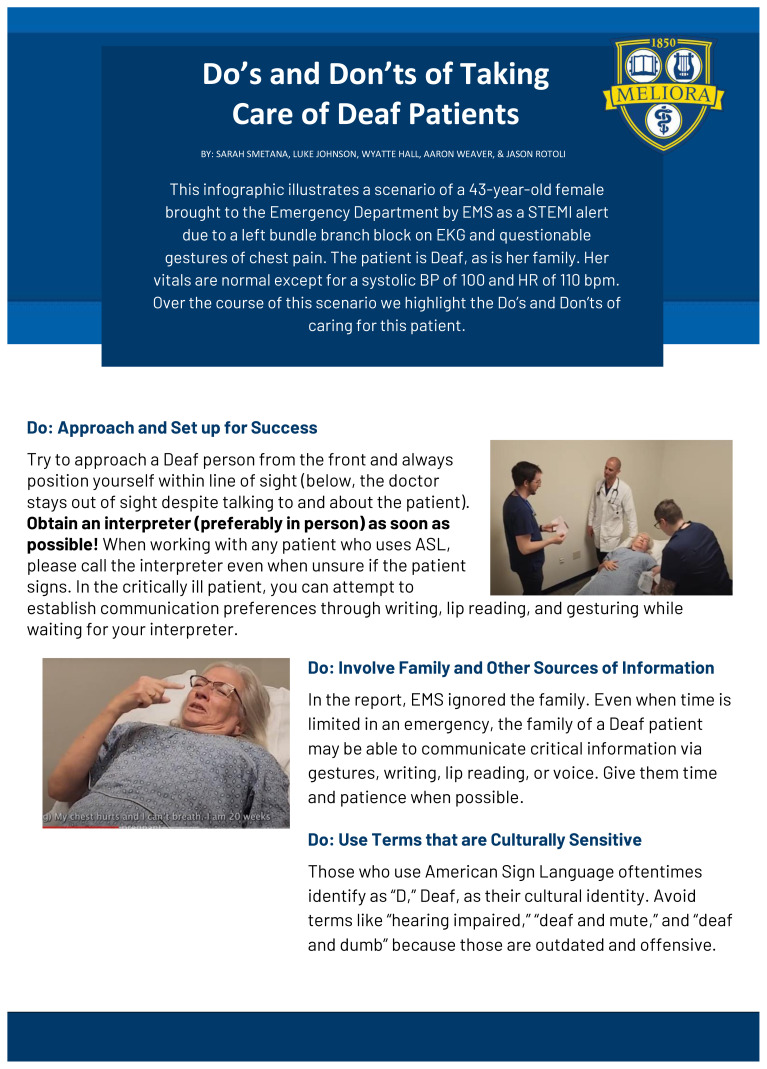


**Figure:**
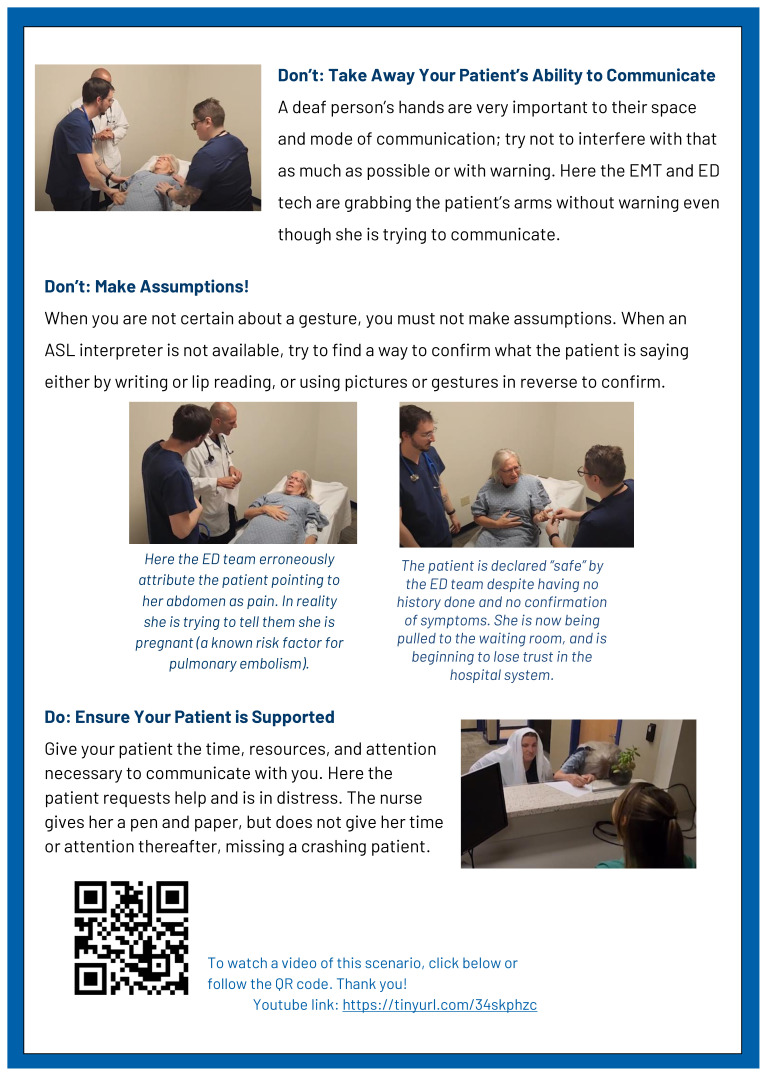


## References

[1] US Census Bureau (2001). Panel Questionnaires. Census.gov.

[2] Mitchell RE (2006). How many deaf people are there in the United States? Estimates from the survey of income and program participation. J Deaf Stud Deaf Educ.

[3] Schein JD, Delk MT (1974). The Deaf Population of the United States.

[4] Mitchell RE, Young TA, Bachleda B, Karchmer MA (2006). How many people use ASL in the United States? Why estimates need updating. Sign Lang Stud.

[5] McKee MM, Winters PC, Sen A, Zazove P, Fiscella K (2015). Emergency department utilization among deaf American Sign Language users. Disabil Health J.

[6] James TG, McKee MM, Miller MD (2022). Emergency department utilization among deaf and hard-of-hearing patients: A retrospective chart review. Disabil Health J.

[7] Conner KR, Jones CM, Wood N (2023). Use of routine emergency department care practices with deaf American Sign Language users. J Emerg Med.

[8] Rotoli J, Li T, Kim S (2020). Emergency department testing and disposition of deaf American Sign Language users and Spanish-speaking patients. J Health Disparities Res Pract.

[9] Wang T (2009). Educational benefits of multimedia skills training. 2009 International Association of Computer Science and Information Technology - Spring Conference.

[10] Mayer RE (2014). Incorporating motivation into multimedia learning. Learn Instr.

[11] Guinane CS, Sikes JI, Wilson RK (1994). Using the PDSA Cycle to standardize a quality assurance program in a quality improvement-driven environment. Jt Comm J Qual Improv.

[12] Harris PA, Taylor R, Thielke R, Payne J, Gonzalez N, Conde JG (2008). Research Electronic Data Capture (REDCap) - A metadata-driven methodology and workflow process for providing translational research informatics support. J Biomed Inform.

[13] Hoang L, LaHousse SF, Nakaji MC, Sadler GR (2011). Assessing deaf cultural competency of physicians and medical students. J Cancer Educ.

[14] Lapinski J, Colonna C, Sexton P, Richard M (2015). American sign language and deaf culture competency of osteopathic medical students. Am Ann Deaf.

[15] Crowfoot D, Prasad Vibhore Using the Plan–Do–Study–Act (PDSA) Cycle to make change in general practice.

[16] Fricker Scott S, Yan Ting Shiao-Lin, Shirley T Response burden: What predicts it and who is burdened out?.

[17] Eysenbach G (2005). The Law of Attrition. J Med Internet Res.

