# Supplementary material for: Do’s and Don’ts of Taking Care of Deaf Patients
Source: J Educ Teach Emerg Med. 2025 Jan 31;10(1):L1–8. doi: 10.21980/J8336T (PMC12096897; doi:10.21980/J8336T)
Supplement: Supplementary file 2 [file 10-1-L1-Supp2.pdf]

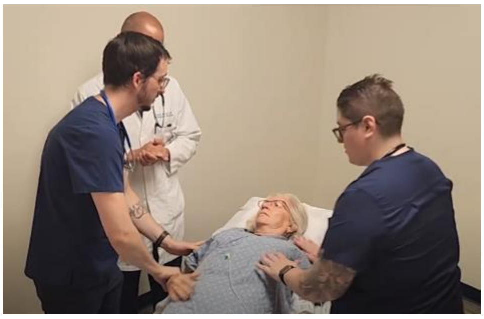

Don’t: Take Away Your Patient’s Ability to Communicate

A deaf person’s hands are very important to their space and mode of communication; try not to interfere with that as much as possible or with warning. Here the EMT and ED tech are grabbing the patient’s arms without warning even though she is trying to communicate.

Don’t: Make Assumptions!

When you are not certain about a gesture, you must not make assumptions. When an ASL interpreter is not available, try to find a way to confirm what the patient is saying either by writing or lip reading, or using pictures or gestures in reverse to confirm.

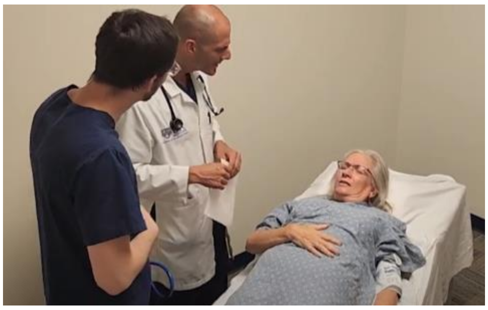

Here the ED team erroneously attribute the patient pointing to her abdomen as pain. In reality she is trying to tell them she is pregnant (a known risk factor for pulmonary embolism).

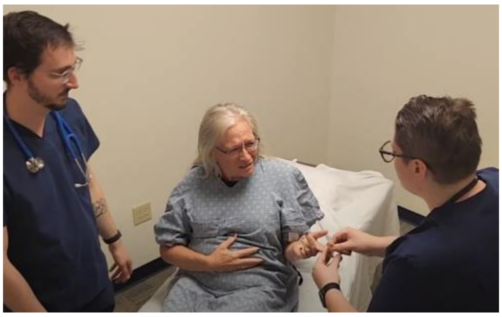

The patient is declared “safe” by the ED team despite having no history done and no confirmation of symptoms. She is now being pulled to the waiting room, and is beginning to lose trust in the hospital system.

Do: Ensure Your Patient is Supported

Give your patient the time, resources, and attention necessary to communicate with you. Here the patient requests help and is in distress. The nurse gives her a pen and paper, but does not give her time or attention thereafter, missing a crashing patient.

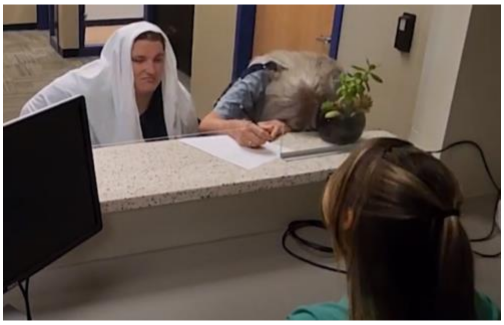

To watch a video of this scenario, click below or follow the QR code. Thank you!

Youtube link: https://tinyurl.com/34skphzc
