# Supplementary material for: Do’s and Don’ts of Taking Care of Deaf Patients
Source: J Educ Teach Emerg Med. 2025 Jan 31;10(1):L1–8. doi: 10.21980/J8336T (PMC12096897; doi:10.21980/J8336T)
Supplement: Supplementary file 4 [file 10-1-L1-Supp4.pdf]

Do’s and Don’ts of Taking Care of Deaf Patients

BY: SARAH SMETANA, LUKE JOHNSON, WYATTE HALL, AARON WEAVER, & JASON ROTOLI

This infographic illustrates a scenario of a 43-year-old female brought to the Emergency Department by EMS as a STEMI alert due to a left bundle branch block on EKG and questionable gestures of chest pain. The patient is Deaf, as is her family. Her vitals are normal except for a systolic BP of 100 and HR of 110 bpm. Over the course of this scenario we highlight the Do’s and Don’ts of caring for this patient.

Do: Approach and Set up for Success

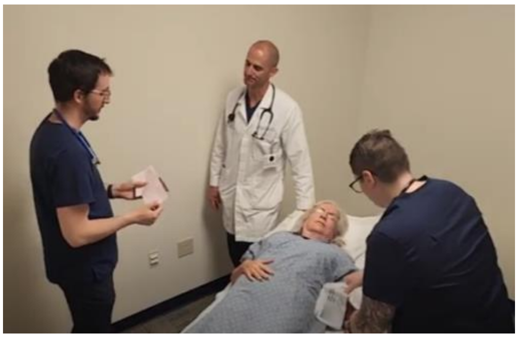

Try to approach a Deaf person from the front and always position yourself within line of sight (below, the doctor stays out of sight despite talking to and about the patient). **Obtain an interpreter (preferably in person) as soon as possible!** When working with any patient who uses ASL, please call the interpreter even when unsure if the patient signs. In the critically ill patient, you can attempt to establish communication preferences through writing, lip reading, and gesturing while waiting for your interpreter.

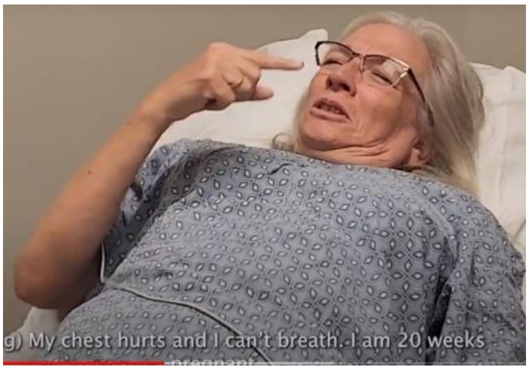

Do: Involve Family and Other Sources of Information

In the report, EMS ignored the family. Even when time is limited in an emergency, the family of a Deaf patient may be able to communicate critical information via gestures, writing, lip reading, or voice. Give them time and patience when possible.

Do: Use Terms that are Culturally Sensitive

Those who use American Sign Language oftentimes identify as “D,” Deaf, as their cultural identity. Avoid terms like “hearing impaired,” “deaf and mute,” and “deaf and dumb” because those are outdated and offensive.

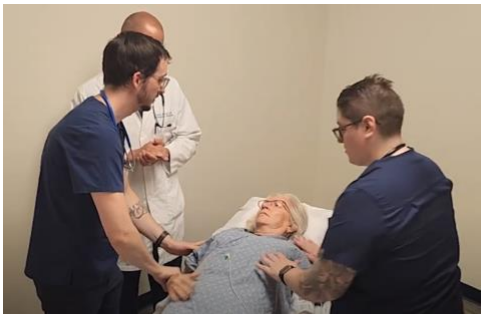

Don't: Take Away Your Patient's Ability to Communicate

A deaf person's hands are very important to their space and mode of communication; try not to interfere with that as much as possible or with warning. Here the EMT and ED tech are grabbing the patient's arms without warning even though she is trying to communicate.

Don't: Make Assumptions!

When you are not certain about a gesture, you must not make assumptions. When an ASL interpreter is not available, try to find a way to confirm what the patient is saying either by writing or lip reading, or using pictures or gestures in reverse to confirm.

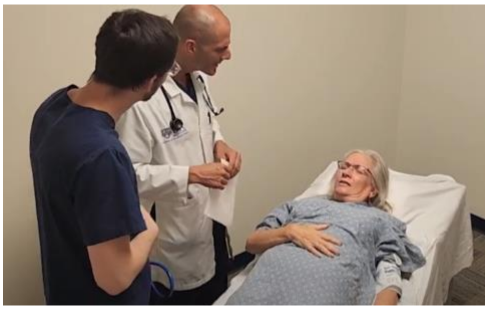

Here the ED team erroneously attribute the patient pointing to her abdomen as pain. In reality she is trying to tell them she is pregnant (a known risk factor for pulmonary embolism).

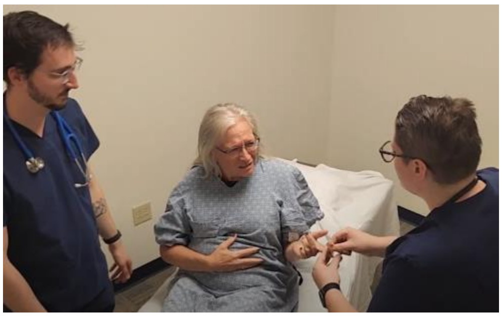

The patient is declared "safe" by the ED team despite having no history done and no confirmation of symptoms. She is now being pulled to the waiting room, and is beginning to lose trust in the hospital system.

Do: Ensure Your Patient is Supported

Give your patient the time, resources, and attention necessary to communicate with you. Here the patient requests help and is in distress. The nurse gives her a pen and paper, but does not give her time or attention thereafter, missing a crashing patient.

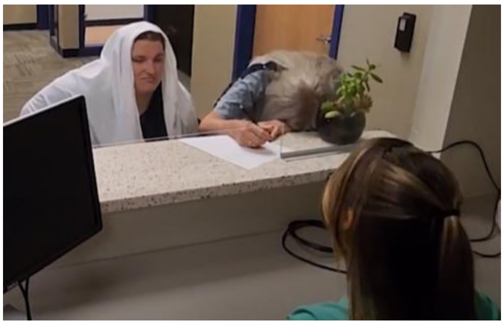

To watch a video of this scenario, click below or follow the QR code. Thank you!

Youtube link: https://tinyurl.com/34skphzc
